# Frequency, characteristics and risk factors of QT interval prolonging drugs and drug-drug interactions in cancer patients: a multicenter study

**DOI:** 10.1186/s40360-017-0181-2

**Published:** 2017-12-01

**Authors:** Qasim Khan, Mohammad Ismail, Sehrash Khan

**Affiliations:** 1 Department of Pharmacy, University of Peshawar, Peshawar, Khyber Pakhtunkhwa Pakistan; 2 Department of Pharmacy, COMSATS Institute of Information Technology, Abbottabad, Pakistan

**Keywords:** QT prolongation, QT prolonging drugs, QT drug-drug interactions, Torsades de pointes, Cancer, Oncology

## Abstract

**Background:**

Cancer patients may receive a high number of medications with the potential to prolong QT interval and subsequent TdP (torsades de pointes). This study aimed to identify the prevalence of QT prolonging drugs, their TdP risk, QT prolonging drug-drug interactions (QT-DDIs), levels, predictors, and TdP risk of drugs involved in QT-DDIs.

**Methods:**

This multicenter study included cancer patients from three major tertiary care hospitals of Khyber-Pakhtunkhwa, Pakistan. Micromedex DrugReax® was used for identification of QT-DDIs. TdP risks were identified by AZCERT (Arizona Center for Education and Research on Therapeutics) classification. Logistic regression analysis was performed to identify predictors of QT-DDIs.

**Results:**

Of 555 patients, 51% were females. Mean age was 46.9 ± 15.7 years. Total 28 distinct QT prolonging drugs were identified in 92.6% of the patients. Overall 21.8% patients were presented with QT-DDIs. Of total 288 identified QT-DDIs, all were of major-severity and fair-documentation. According to AZCERT classification, 59.9% of the interacting drugs were included in list-1 (known risk of TdP), 4.7% in list-2 (possible risk of TdP) and 6.8% in list-3 (conditional risk of TdP). Univariate logistic regression analysis showed significant results for various predictors such as, 8–9 prescribed medications (*p* < 0.001) and ≥10 medications (*p* < 0.001), 2 QT drugs (*p* < 0.001) and ≥3 QT drugs (*p* < 0.001), breast cancer (*p* = 0.03), gastrointestinal cancer (*p* = 0.03), 4–5 supportive care drugs (*p* < 0.001), 6–8 supportive care drugs (*p* < 0.001) and >8 supportive care drugs (*p* < 0.001).

**Conclusions:**

A high prevalence of QT prolonging drugs and QT-DDIs was reported in oncology. Appropriate precautions are needed to prevent harmful consequences of these interactions.

**Electronic supplementary material:**

The online version of this article (10.1186/s40360-017-0181-2) contains supplementary material, which is available to authorized users.

## Background

In developed world, cancer and cardiac disease play a major role in causing morbidity and mortality [1]. Due to recent therapeutic advancements, 5-year survival for early stage breast cancer increased from 79% to 88% during the last two decades [2–5]. Similarly, survival rates have also been increased in other solid and hematological cancers as well as non-hodgkin lymphoma and testicular cancer [6].

Numerous drugs are administered to the patients with advanced cancer in order to treat their malignancy, its related ailments (e.g., pain), comorbid illnesses (e.g., heart disease, diabetes, dyslipidemia), and mitigate adverse effects induced by chemotherapy (e.g., nausea and vomiting). Certainly, multiple therapies make cancer patients vulnerable to potentially unsafe drug-drug interactions (DDIs) and it can be worsened in the presence of aberrant organ function (heart, liver, and kidney) [7].

Since last few years, cancer patients have been predisposed to substantial medical complications in the form of heart diseases [8]. A distinctive range of cardiovascular anomalies including, myocardial toxicity, ischemia, hypertension and arrhythmia [9–13] either directly or indirectly (inappropriate lifestyle) have been associated with new cancer therapies [1]. Moreover, anticancer agents and supportive care therapy may cause various cardiac rhythm disorders and most remarkable feature is prolonged QT interval which can ultimately lead to ventricular arrhythmias. Concomitant use of supportive care therapy and cancer medications may cause prolongation of QT interval [1].

The QT interval on an electrocardiography (ECG) rhythm strip indicates phases of ventricular depolarization and consequent repolarization and its measurement is taken from the point where QRS complex begins to the end of T wave [7]. A delay in the cardiac repolarization phase leads to the electrophysiological disturbances and subsequent torsades de pointes (TdP) [14, 15]. TdP is a rare form of fatal polymorphic ventricular tachycardia that is often illustrated by the twisting of points on an ECG [7]. Currently, pharmacoepidemiologic data regarding prevalence and nature of QT prolonging drug-drug interactions (QT-DDIs) in cancer patients is limited and there are certain areas which need to be explored. Issue of QT-DDIs in cancer patients is a poorly addressed area. To the best of our knowledge, there is no specific study regarding the prevalence of QT-DDIs in oncology settings. There are some studies which have worked on the prevalence and nature of overall potential DDIs in cancer patients [16–18]. As the main aims of these studies were to explore all types of DDIs in a generalized manner, therefore limited considerations have been given to QT-DDIs. All of these studies have elaborated in their discussions that proper attention should be given to QT-DDIs and their associated negative consequences in cancer patients [16–18]. Therefore, specific work is needed in cancer patients to explore the prevalence of QT-DDIs, possible risk factors, extent of the risk of QTc prolongation and possible predictors. Lack of scientific evidence regarding prescribing pattern of QT prolonging medications, QT-DDIs and QTc prolongation may predispose cancer patients to TdP. Such studies will be helpful to improve clinical practice and ensure patients’ safety.

### Aim of the study

The aim of this study was to investigate the frequency of QT prolonging drugs and their TdP risk; and QT-DDIs, their levels of severity and documentation, predictors and TdP risk of drugs involved in QT-DDIs.

## Methods

### Study design and settings

This was a multicenter cross-sectional retrospective study conducted in three tertiary care hospitals, Medical Teaching Institute, Ayub Teaching Hospital (ATH), Abbottabad, North West General Hospital and Research Center, Peshawar and Medical Teaching Institute, Hayatabad Medical Complex (HMC), Peshawar, Pakistan.

### Data source

The study included data of all consecutive patients, aged >18 years, who received treatment for cancer during a one-year period, Jan-2014 to Dec-2014. Approval was obtained from hospitals’ administrations to access patients’ data in order to collect all relevant information needed for the study. Data were collected regarding patients’ age, gender, cancer type, comorbidities and prescribed medications.

### Data analysis

For each patient, medication lists were analyzed for the presence of QT-DDIs using an online database, Micromedex Drug-Reax® [19]. QT-DDIs were classified on the basis of severity and documentation according to the Micromedex Drug-Reax® classification system [19]. The Arizona Center for Education and Research on Therapeutics (AZCERT) QT drug list [20] was used for identifying QT prolonging drugs. The AZCERT classification system categorizes QT prolonging drugs in to list-1 (known risk of TdP), list-2 (possible risk of TdP), and list-3 (conditional risk of TdP). Therapeutic classes of drugs involved in QT-DDIs were coded according to Anatomical Therapeutic Chemical (ATC) index of the World Health Organization (WHO) [21].

### Statistical analyses

Categorical data were presented as frequencies and percentages. While continuous data were presented as mean ± SD. Logistic regression analysis was used to calculate the odds ratios (OR) for predictors of QT-DDIs. A *p*-value ≤0.05 was considered statistically significant. SPSS (IBM SPSS statistics version 23) was used for all statistical analyses.

## Results

Patients’ demographic and clinical characteristics are listed in Table 1. Total 555 patients were included in this study, of which 274 (49%) were males and 281 (51%) were females. Mean age of the patients was 46.9 ± 15.7 years, whereas majority of the patients were in the age range > 50 years (39.5%). Average number of prescribed medications were 8.4 ± 3.6, while in 35.9% of the cases, ≥10 drugs were prescribed. The most frequent diagnoses were breast cancer (15.3%), non-hodgkin lymphoma (15.1%), gastrointestinal cancer (12.8%), gynecologic cancer (5.9%), acute lymphoblastic leukemia (5.2%), and genitourinary cancer (4.1%). The most frequent comorbid illnesses were diabetes mellitus (4.9%), hypertension (4.1%), hepatitis B (0.5%) and hepatitis C (0.5%).

**Table 1:** Patients’ demographic and clinical characteristics

Variable	Patients: n (%)^a^
Gender	
Male	274 (49)
Female	281 (51)
Age	
≤ 30	120 (21.6)
31–40	94 (16.9)
41–50	122 (22)
> 50	219 (39.5)
Overall prescribed drugs^b^	
≤ 5	111 (20)
6–7	114 (20.5)
8–9	131 (23.6)
≥ 10	199 (35.9)
Diagnoses	
Breast cancer	86 (15.5)
Non hodgkin lymphoma	84 (15.1)
Gastrointestinal cancer	71 (12.8)
Gynecologic cancer	33 (5.9)
Acute lymphoblastic leukemia	29 (5.2)
Genitourinary cancer	23 (4.1)
Hodgkin lymphoma	17 (3.1)
Chronic lymphocytic leukemia	15 (2.7)
Musculoskeletal cancer	14 (2.5)
Acute mylogenous leukemia	11 (2)
Ovarian cancer	11 (2)
Colorectal carcinoma	11 (2)
Lung cancer	10 (1.8)
Head and neck cancer	8 (1.4)
Comorbidities	
Diabetes mellitus	27 (4.9)
Hypertension	23 (4.1)
Hepatitis B	3 (0.5)
Hepatitis C	3 (0.5)

^a^Percentage calculated in total number of patients i.e., 555

^b^Overall prescribed medications mean QT prolonging medications as well as other medications

Total 993 QT prolonging drugs were identified in 92.6% of the patients (Table 2). Among them 46.5% were females while 46.1% were males. The cancer patients were frequently encountered with antiemetics (*n* = 571), proton pump inhibitors (145), antimicrobials (126), anticancer drugs (51) and antineoplastic agents (30) which carried the potential for QT prolongation (Table 2). Total 28 distinct QT prolonging drugs were used in cancer patients. Among them, the most prevalent were ondansetron (*n* = 278), metoclopramide (152), tropisetron (139), ciprofloxacin (90), omeprazole (87), capecitabine (46) and oxaliplatin (30).

**Table 2:** Prevalence of the QT interval prolonging drugs with their therapeutic classes and TdP risks

Prevalence/ classification scheme			Frequency
			Patients: *n* (%)^a^
Overall prevalence of the QT prolonging drugs			514 (92.6)
Gender-wise prevalence of QT prolonging drugs			
Male			256 (46.1)
Female			258 (46.5)
Therapeutic class	TdP risk^b^	QT drug (ATC code)	Patients: *n* (%)^a^
Antimicrobials (*n* = 126)	Known risk of TdP (*n* = 98)	Ciprofloxacin (J01MA02)	90 (16.2)
		Clarithromycin (J01FA09)	6 (1)
		Levofloxacin (J01MA12)	1 (0.2)
		Moxifloxacin (J01MA14)	1 (0.2)
	Possible risk of TdP (*n* = 1)	Norfloxacin (J01MA06)	1 (0.2)
	Conditional risk of TdP (*n* = 27)	Metronidazole (P01AB01)	27 (4.9)
Anticancer (*n* = 51)	Possible risk of TdP (*n* = 51)	Capecitabine (L01 BC06)	46 (8.3)
		Tamoxifen (L02BA01)	5 (0.9)
Antidepressant (*n* = 4)	Conditional risk of TdP (*n* = 4)	Amitriptyline (N06AA09)	3 (0.5)
		Fluoxetine (N06AB03)	1 (0.2)
Antidiarrheal (*n* = 1)	Conditional risk of TdP (*n* = 1)	Loperamide (A07DA03)	1 (0.2)
Antiemetic (*n* = 571)	Known risk of TdP (*n* = 278)	Ondansetron (A04AA01)	278 (50)
	Possible risk of TdP (*n* = 141)	Tropisetron (A04AA03)	139 (25)
		Promethazine (R06AD02)	2 (0.4)
	Conditional risk of TdP (*n* = 152)	Metoclopramide (A03FA01)	152 (27.4)
Antifungal (*n* = 11)	Known risk of TdP (*n* = 6)	Fluconazole (J02 AC01)	6 (1)
	Conditional risk of TdP (*n* = 5)	Amphotericin B (J02AA01)	3 (0.5)
		Ketoconazole (J02AB02)	2 (0.4)
Antihistamine (*n* = 8)	Conditional risk of TdP (*n* = 8)	Diphenhydramine (R06AA52)	8 (1.1)
Antinausea (*n* = 21)	Known risk of TdP (*n* = 21)	Domperidone (A03FA03)	21 (3.8)
Antineoplastic (*n* = 30)	Known risk of TdP (*n* = 30)	Oxaliplatin (L01XA03)	30 (5.4)
Antipsychotic (*n* = 1)	Known risk of TdP (*n* = 1)	Haloperidol (N05 AD01)	1 (0.2)
Diuretic (*n* = 22)	Conditional risk of TdP (*n* = 22)	Furosemide (C03CA01)	19 (3.4)
		Hydrochlorothiazide (C03AX01)	3 (0.5)
Gonadotropin receptor agonist/antagonist (*n* = 1)	Possible risk of TdP (*n* = 1)	Leuprolide (L02AE02)	1 (0.2)
Kinase inhibitor (*n* = 1)	Possible risk of TdP (*n* = 1)	Nilotinib (L01XE08)	1 (0.2)
Proton pump inhibitor (*n* = 145)	Conditional risk of TdP (*n* = 145)	Esomeprazole (A02BC05)	59 (10.6)
		Omeprazole (A02BC01)	86 (15.5)

*AZCERT* Arizona Center for Education and Research on Therapeutics, *TdP* torsades de pointes

^a^Percentage calculated in total number of patients i.e., 555

^b^TdP risk was based on the AZCERT QT drugs lists

Table 3 shows the highly prevalent (*n* > 5) QT prolonging drugs used in various types of cancer, such as, breast cancer: ondansetron (54), ciprofloxacin (32), tropisetron (20), and metoclopramide (18); gastrointestinal cancer: ondansetron (46), capecitabine (25), metoclopramide (21), and oxaliplatin (20); and non-hodgkin lymphoma: ondansetron (39), tropisetron (33), metoclopramide (25), and esomeprazole (16). A full presentation of all QT prolonging drugs stratified with respect to various types of cancer has been given in Additional file 1: Table S1.

**Table 3:** Highly prevalent QT interval prolonging drugs (≥5)^a^ in various types of cancer

QT drugs	TdP risk^b^	QT drugs: *n* (%)^c^
Breast cancer		
Ondansetron	Known risk of TdP	54 (5.4)
Ciprofloxacin	Known risk of TdP	32 (3.2)
Tropisetron	Possible risk of TdP	20 (2)
Metoclopramide	Conditional risk of TdP	18 (1.8)
Omeprazole	Conditional risk of TdP	8 (0.8)
Esomeprazole	Conditional risk of TdP	6 (0.6)
Gastrointestinal cancer		
Ondansetron	Known risk of TdP	46 (4.6)
Capecitabine	Possible risk of TdP	25 (2.5)
Metoclopramide	Conditional risk of TdP	21 (2.1)
Oxaliplatin	Known risk of TdP	20 (2)
Tropisetron	Possible risk of TdP	17 (1.7)
Omeprazole	Conditional risk of TdP	17 (1.7)
Ciprofloxacin	Known risk of TdP	13 (1.3)
Esomeprazole	Conditional risk of TdP	7 (0.7)
Non hodgkin lymphoma		
Ondansetron	Known risk of TdP	39 (3.9)
Tropisetron	Possible risk of TdP	33 (3.3)
Metoclopramide	Conditional risk of TdP	25 (2.5)
Esomeprazole	Conditional risk of TdP	16 (1.6)
Omeprazole	Conditional risk of TdP	14 (1.4)
Ciprofloxacin	Known risk of TdP	5 (0.5)
Gynecologic cancer		
Ondansetron	Known risk of TdP	25 (2.5)
Tropisetron	Possible risk of TdP	9 (0.9)
Ciprofloxacin	Known risk of TdP	8 (0.8)
Metoclopramide	Conditional risk of TdP	7 (0.7)
Omeprazole	Conditional risk of TdP	7 (0.7)
Esomeprazole	Conditional risk of TdP	5 (0.5)
Genitourinary cancer		
Ondansetron	Known risk of TdP	18 (1.8)
Esomeprazole	Conditional risk of TdP	5 (0.5)
Acute lymphoblastic leukemia		
Metoclopramide	Conditional risk of TdP	16 (1.6)
Omeprazole	Conditional risk of TdP	16 (1.6)
Ondansetron	Known risk of TdP	7 (0.7)
Metronidazole	Conditional risk of TdP	6 (0.6)
Chronic lymphocytic leukemia		
Tropisetron	Possible risk of TdP	11 (1.1)
Metoclopramide	Conditional risk of TdP	8 (0.8)
Hodgkin lymphoma		
Ondansetron	Known risk of TdP	9 (0.9)
Metoclopramide	Conditional risk of TdP	8 (0.8)
Musculoskeletal cancer		
Ondansetron	Known risk of TdP	9 (0.9)
Metoclopramide	Conditional risk of TdP	7 (0.7)
Tropisetron	Possible risk of TdP	6 (0.6)
Colorectal carcinoma		
Capecitabine	Possible risk of TdP	8 (0.8)
Oxaliplatin	Known risk of TdP	7 (0.7)
Ondansetron	Known risk of TdP	5 (0.5)
Acute mylogenous leukemia		
Omeprazole	Conditional risk of TdP	7 (0.7)
Metoclopramide	Conditional risk of TdP	5 (0.5)
Lung cancer		
Ondansetron	Known risk of TdP	6 (0.6)
Neurological cancer		
Ondansetron	Known risk of TdP	6 (0.6)
Adenocarcinoma		
Metoclopramide	Conditional risk of TdP	5 (0.5)

*AZCERT* Arizona Center for Education and Research on Therapeutics, *TdP* torsades de pointes

^a^Remaining results have been mentioned in Additional file 1: Table S1

^b^TdP risk was based on the AZCERT QT drugs lists

^c^Percentage calculated in total number of QT interval prolonging drugs i.e., 993

**Fig. 1:**
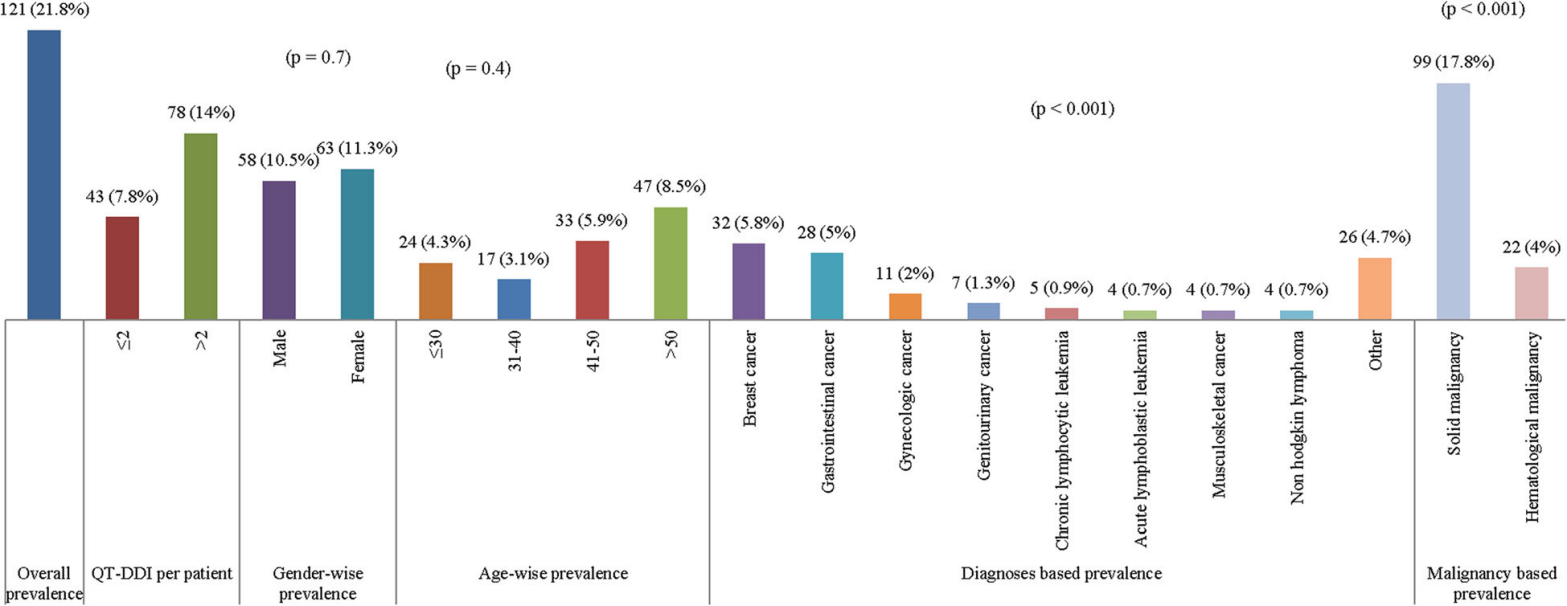
Prevalence of QT drug-drug interactions (QT-DDIs) stratified with respect to gender, age, diagnosis and types of malignancy

Of 555 patients, 21.8% were presented with QT-DDIs (Fig. 1). Prevalence of QT-DDIs was higher in females (11.3%) as compared with males (10.5%) (*p* = 0.7) and in age group >50 years (8.5%) as compared with other age groups (*p* = 0.4). Similarly, prevalence of QT-DDIs was significantly higher in breast cancer (5.8%) and gastrointestinal cancer (5%) compared with other cancers (*p* < 0.001) and in solid malignancy (17.8%) compared with hematological malignancy (4%) (p < 0.001) (Fig. 1).

Total 288 QT-DDIs were identified, of which, all were of major severity and fair documentation (Table 4). According to AZCERT classification, 59.9% of the interacting drugs were included in list-1 (known risk of TdP), 4.7% in list-2 (possible risk of TdP) and 6.8% in list-3 (conditional risk of TdP) (Table 4). As far as therapeutic classes are concerned, antimicrobials (36.3%), antiemetic (34.7%) and antipsychotics (27.3%) were more common. Table 5 shows top 20 QT-DDIs, their AZCERT classification, [20] therapeutic classes, severity and documentation levels. Of the total QT-DDIs, 76 QT-DDIs involved both the interacting drugs from the AZCERT QT drugs list-1 (known risk of TdP). The most common drug interacting pairs involved in QT-DDIs were ondansetron-prochlorperazine (*n* = 88), ciprofloxacin-ondansetron (71), ciprofloxacin-prochlorperazine (64), ciprofloxacin-metronidazole (10) and ciprofloxacin-dolasetron (6). Drugs frequently involved in QT-DDIs were ondansetron (*n* = 174), ciprofloxacin (157), prochlorperazine (157), metronidazole (30), dolasetron (21) and fluconazole (8).

**Table 4:** Prevalence of the QT-DDIs, TdP risk, therapeutic classes, severity and documentation of drugs involved in QT DDIs

Classification scheme	Interacting drugs: *n* (%)^a^
TdP risk^b^	
Known risk of TdP (List 1)	345 (59.9)
Possible risk of TdP (List 2)	27 (4.7)
Conditional risk of TdP (List 3)	39 (6.8)
Not included in AZCERT QT drug list (List 4)	165 (28.6)
Therapeutic classes (ATC code)	
Antimicrobial (J)	209 (36.3)
Antiemetic (A04)	200 (34.7)
Antipsychotic (N05A)	157 (27.3)
Muscle relaxant (M03)	3 (0.5)
Kinase inhibitor (L01XE)	3 (0.5)
Antidiarrheal (A)	2 (0.4)
Anticancer (L01)	2 (0.4)
Antidepressant (N06A)	2 (0.4)
Classification on the basis of severity^c^	QT-DDIs: *n* (%)^d^
Major	288 (100)
Classification on the basis of documentation^c^	QT-DDIs: *n* (%)^d^
Fair	288 (100)

*AZCERT* Arizona Center for Education and Research on Therapeutics, *TdP* torsades de pointes, *QT DDIs* QT prolonging drug-drug interactions, *ATC* Anatomical Therapeutic Chemical

^a^Percentage calculated in number of all interacting drugs i.e., 576

^b^TdP risk was based on the AZCERT QT drugs lists

^c^Severity and documentation were based on Micromedex DrugReax classification

^d^Percentage calculated in total number of QT-DDIs i.e., 288

**Table 5:** Top 20 QT drug-drug interaction (QT-DDI) along with their levels, therapeutic class and TdP risks of drugs involved in QT-DDIs

QT-DDIs	Therapeutic class		TdP risk^a^		Levels of QT-DDIs^b^		Frequency
	Drug 1	Drug 2	Drug 1	Drug 2	Severity	Documentation	QT-DDIs: *n* (%)^c^
Ondansetron-Prochlorperazine	Antiemetic	Antipsychotic	Known risk of TdP	Not included in lists^d^	Major	Fair	88 (30.6)
Ciprofloxacin-Ondansetron	Antimicrobial	Antiemetic	Known risk of TdP	Known risk of TdP	Major	Fair	71 (24.7)
Ciprofloxacin-Prochlorperazine	Antimicrobial	Antipsychotic	Known risk of TdP	Not included in lists	Major	Fair	64 (22.2)
Ciprofloxacin-Metronidazole	Antimicrobial	Antimicrobial	Known risk of TdP	Conditional risk of TdP	Major	Fair	10 (3.5)
Ciprofloxacin-Dolasetron	Antimicrobial	Antiemetic	Known risk of TdP	Possible risk of TdP	Major	Fair	6 (2.1)
Dolasetron -Ondansetron	Antiemetic	Antiemetic	Possible risk of TdP	Known risk of TdP	Major	Fair	5 (1.7)
Dolasetron-Metronidazole	Antiemetic	Antimicrobial	Possible risk of TdP	Conditional risk of TdP	Major	Fair	5 (1.7)
Metronidazole-Ondansetron	Antimicrobial	Antiemetic	Conditional risk of TdP	Known risk of TdP	Major	Fair	4 (1.4)
Fluconazole-Metronidazole	Antimicrobial	Antimicrobial	Known risk of TdP	Conditional risk of TdP	Major	Fair	3 (1)
Prochlorperazine-Promethazine	Antipsychotic	Antiemetic	Not included in lists	Possible risk of TdP	Major	Fair	2 (0.7)
Ondansetron-Promethazine	Antiemetic	Antiemetic	Known risk of TdP	Conditional risk of TdP	Major	Fair	2 (0.7)
Metronidazole-Nilotinib	Antimicrobial	Kinase inhibitor	Conditional risk of TdP	Conditional risk of TdP	Major	Fair	2 (0.7)
Dolasetron-Octreotide	Antiemetic	Antidiarrheal	Possible risk of TdP	Not included in lists	Major	Fair	2 (0.7)
Clarithromycin-Ondansetron	Antimicrobial	Antiemetic	Known risk of TdP	Known risk of TdP	Major	Fair	2 (0.7)
Ciprofloxacin-Ketoconazole	Antimicrobial	Antimicrobial	Known risk of TdP	Conditional risk of TdP	Major	Fair	2 (0.7)
Ciprofloxacin-Fluconazole	Antimicrobial	Antimicrobial	Known risk of TdP	Known risk of TdP	Major	Fair	2 (0.7)
Prochlorperazine-Trimethoprim	Antipsychotic	Antimicrobial	Not included in lists	Not included in lists	Major	Fair	1 (0.3)
Prochlorperazine-Sulfamethoxazole	Antipsychotic	Antimicrobial	Not included in lists	Not included in lists	Major	Fair	1 (0.3)
Ondansetron-Tizanidine	Antiemetic	Muscle relaxant	Known risk of TdP	Not included in lists	Major	Fair	1 (0.3)
Nilotinib-Tizanidine	Kinase inhibitor	Muscle relaxant	Conditional risk of TdP	Not included in lists	Major	Fair	1 (0.3)
Metronidazole-Tizanidine	Antimicrobial	Muscle relaxant	Conditional risk of TdP	Not included in lists	Major	Fair	1 (0.3)
Metronidazole-Prochlorperazine	Antimicrobial	Antipsychotic	Conditional risk of TdP	Not included in lists	Major	Fair	1 (0.3)
Metronidazole-Norfloxacin	Antimicrobial	Antimicrobial	Conditional risk of TdP	Possible risk of TdP	Major	Fair	1 (0.3)

*AZCERT* Arizona Center for Education and Research on Therapeutics, *TdP* torsades de pointes, *QT DDIs* QT prolonging drug-drug interactions

^a^TdP risk was based on AZCERT QT drugs list

^b^Levels i.e., severity and documentation were based on Micromedex DrugReax® classification

^c^Percentage calculated in total number of QT-DDIs i.e., 288

^d^Drugs involved in QT-DDIs were not included in the AZCERT QT drugs lists

Table 6 shows the highly prevalent (*n* > 2) QT-DDIs in various types of cancer, such as, breast cancer: ciprofloxacin-Ondansetron (31), ciprofloxacin-prochlorperazine (31), and ondansetron-prochlorperazine (31); gastrointestinal cancer: ondansetron-prochlorperazine (26), ciprofloxacin-ondansetron (13), and ciprofloxacin-prochlorperazine (12); and gynecologic cancer: ciprofloxacin-ondansetron (8), ciprofloxacin-prochlorperazine (6), and Ondansetron-Prochlorperazine (6). The entire result has been provided in Additional file 2: Table S2 which shows frequency of all QT-DDIs along with their levels and TdP risks of drugs involved in these QT-DDIs stratified with respect to various types of cancer.

**Table 6:** The most frequent (≥2)^a^ QT-DDIs along with their levels and TdP risks of drugs involved in these QT-DDIs stratified with respect to various types of cancer

QT-DDIs	TdP risk		Levels of QT-DDIs		Frequency
	Drug 1	Drug 2	Severity	Documentation	QT-DDIs: *n* (%)^b^
Breast cancer					
Ciprofloxacin-Ondansetron	Known risk of TdP	Known risk of TdP	Major	Fair	31 (10.8)
Ciprofloxacin-Prochlorperazine	Known risk of TdP	Not included in lists	Major	Fair	31 (10.8)
Ondansetron-Prochlorperazine	Known risk of TdP	Not included in lists	Major	Fair	31 (10.8)
Gastrointestinal cancer					
Ondansetron-Prochlorperazine	Known risk of TdP	Not included in lists	Major	Fair	26 (9)
Ciprofloxacin-Ondansetron	Known risk of TdP	Known risk of TdP	Major	Fair	13 (4.5)
Ciprofloxacin-Prochlorperazine	Known risk of TdP	Not included in lists	Major	Fair	12 (4.2)
Dolasetron-Ondansetron	Possible risk of TdP	Known risk of TdP	Major	Fair	2 (0.7)
Gynecologic cancer					
Ciprofloxacin-Ondansetron	Known risk of TdP	Known risk of TdP	Major	Fair	8 (2.8)
Ciprofloxacin-Prochlorperazine	Known risk of TdP	Not included in lists	Major	Fair	6 (2.1)
Ondansetron-Prochlorperazine	Known risk of TdP	Not included in lists	Major	Fair	6 (2.1)
Genitourinary cancer					
Ondansetron-Prochlorperazine	Known risk of TdP	Not included in lists	Major	Fair	7 (2.4)
Musculoskeletal cancer					
Ciprofloxacin-Ondansetron	Known risk of TdP	Known risk of TdP	Major	Fair	4 (1.4)
Ciprofloxacin-Prochlorperazine	Known risk of TdP	Not included in lists	Major	Fair	4 (1.4)
Ondansetron-Prochlorperazine	Known risk of TdP	Not included in lists	Major	Fair	4 (1.4)
Chronic lymphocytic leukemia					
Dolasetron-Metronidazole	Possible risk of TdP	Conditional risk of TdP	Major	Fair	3 (1)
Ciprofloxacin-Dolasetron	Known risk of TdP	Possible risk of TdP	Major	Fair	2 (0.7)
Metronidazole-Nilotinib	Conditional risk of TdP	Conditional risk of TdP	Major	Fair	2 (0.7)
Non hodgkin lymphoma					
Ciprofloxacin-Metronidazole	Known risk of TdP	Conditional risk of TdP	Major	Fair	2 (0.7)
Ciprofloxacin-Ondansetron	Known risk of TdP	Known risk of TdP	Major	Fair	2 (0.7)
Ondansetron-Prochlorperazine	Known risk of TdP	Not included in lists	Major	Fair	2 (0.7)
Adenocarcinoma					
Ciprofloxacin-Ondansetron	Known risk of TdP	Known risk of TdP	Major	Fair	2 (0.7)
Ciprofloxacin-Prochlorperazine	Known risk of TdP	Not included in lists	Major	Fair	2 (0.7)
Ondansetron-Prochlorperazine	Known risk of TdP	Not included in lists	Major	Fair	2 (0.7)
Head and neck cancer					
Ciprofloxacin-Ondansetron	Known risk of TdP	Known risk of TdP	Major	Fair	2 (0.7)
Ciprofloxacin-Prochlorperazine	Known risk of TdP	Not included in lists	Major	Fair	2 (0.7)
Ondansetron-Prochlorperazine	Known risk of TdP	Not included in lists	Major	Fair	2 (0.7)
Neurological cancer					
Ondansetron-Prochlorperazine	Known risk of TdP	Not included in lists	Major	Fair	2 (0.7)
Chronic myelogenous leukemia					
Metronidazole-Nilotinib	Conditional risk of TdP	Conditional risk of TdP	Major	Fair	2 (0.7)

*AZCERT* Arizona Center for Education and Research on Therapeutics, *TdP* torsades de pointes, *QT DDIs* QT prolonging drug-drug interactions

^a^All results have been mentioned in Additional file 2: Table S2

^b^Percentage calculated in total number of QT-DDIs i.e., 288

In univariate logistic regression analysis (Table 7), a significant association of QT-DDIs with 8–9 prescribed medications (OR = 8.9; 95% CI = 2.6–30.3; *p* < 0.001), ≥10 prescribed medications (OR = 25.2; 95% CI = 7.7–82.2; *p* < 0.001), 2 QT prolonging drugs (OR = 25.4; 95% CI = 11.2–57.5; *p* < 0.001) and ≥3 QT prolonging drugs (OR = 21; 95% CI = 9.2–48; *p* < 0.001). There was significant association of the occurrence of QT-DDIs with breast cancer (OR = 3.7; 95% CI = 1.2–11.6; *p* = 0.03), gastrointestinal cancer (OR = 4; 95% CI = 1.3–13; *p* = 0.02), 4–5 supportive care drugs (OR = 4.3; 95% CI = 1.9–9.5; *p* < 0.001), 6–8 supportive care drugs (OR = 8.1; 95% CI = 3.7–17.7; p < 0.001) and >8 supportive care drugs (OR = 12.2; 95% CI = 4.9–30.5; *p* < 0.001).

**Table 7:** Logistic regression analysis

Variables	OR (95% CI)	*p*-value
Gender		
Female	1 (0.7–1.6)	0.7
Age categories		
≤ 30	Reference	
31–40	0.8 (0.4–1.7)	0.6
41–50	1.4 (0.8–2.6)	0.3
> 50	1 (0.6–1.7)	1
Overall prescribed drugs		
≤ 5	Reference	
6–7	3.5 (0.9–12.9)	0.07
8–9	8.9 (2.6–30.3)	<0.001
≥ 10	25.2 (7.7–82.2)	<0.001
QT drugs		
1	Reference	
2	25.4 (11.2–57.5)	<0.001
≥ 3	21 (9.2–48)	<0.001
Diagnoses		
Acute lymphoblastic leukemia	Reference	
Breast cancer	3.7 (1.2–11.6)	0.03
Chronic lymphocytic leukemia	3.5 (0.8–15.9)	0.1
Gastrointestinal cancer	4 (1.3–13)	0.02
Genitourinary cancer	2.7 (0.7–10.9)	0.2
Gynecologic cancer	3.1 (0.9–11.2)	0.08
Musculoskeletal cancer	2.5 (0.5–12)	0.3
Non hodgkin lymphoma	0.3 (0.07–1.3)	0.1
Others	1 (0.3–3.3)	0.9
Anticancer drugs		
≤ 2	Reference	
> 2	0.6 (0.4–0.9)	0.02
Supportive care drugs		
≤ 3	Reference	
4–5	4.3 (1.9–9.5)	<0.001
6–8	8.1 (3.7–17.7)	<0.001
> 8	12.2 (4.9–30.5)	<0.001

## Discussion

This is the first study in oncology which specifically and extensively determined various drug related factors having potential of QT interval prolongation. In this study, we detected a high prevalence of QT prolonging drugs and QT-DDIs, which is of particular concern. Several important findings have emerged from our analysis. The patients with breast cancer and gastrointestinal cancer are at increased risk of TdP due to frequent use of high risk QT interval prolonging medications and QT-DDIs involving both drugs from AZCERT list-1 (known risk of TdP). Proper considerations should be given to monitor the effects of these medications and QT-DDIs in high risk patients. Polypharmacy was the major issue in cancer patients, which might be responsible for such a high prevalence of QT prolonging drugs and QT-DDIs.

The most frequent QT prolonging drugs used in cancer patients were ondansetron, metoclopramide, tropisetron, ciprofloxacin, capecitabine and oxaliplatin which are also responsible for high prevalence of QT-DDIs. While the most common drugs involved in QT-DDIs were ondansetron, metoclopramide, quinolones, capecitabine, oxaliplatin and domperidone. Domperidone is associated with QTc prolongation, subsequent TdP and sudden cardiac death [22]. The published data suggest that ondansetron, metoclopramide and fluoroquinolones may significantly prolong the QT interval causing serious arrhythmias and mortality [23–25]. The monitoring of arrhythmogenic risks associated with these medications is mandatory to avoid life threatening situations.

The data regarding the prevalence of QT interval prolonging drugs and QT-DDIs in oncology settings are scarce. Over the past few years, a limited number of studies investigated the prevalence of QT-DDIs among cancer patients [16–18]. We identified 288 QT-DDIs in contrast to 45–110 QT-DDIs reported by those studies [16–18]. The lack of consistency in results might be due to a variety of reasons. The study design and various tools used for screening QT-DDIs were different. Moreover, the scope and nature of these studies regarding the prevalence of QT-DDIs was limited.

Previous studies [16, 18] screened QT-DDIs using AZCERT drug list, [20] which demonstrates that they considered only pharmacodynamic interactions whereas both pharmacokinetic and pharmacodynamic drug interactions were taken in to account in our analysis. The latest and updated tool, Micromedex Drug-Reax® [19] was used for screening QT-DDIs along with AZCERT QT drug lists [20]. A cross-sectional study considered oral anticancer drugs only while we included all drugs in our study [16]. Variations in prescribing patterns and clinical profile of the patients might me some other factors responsible for these inconsistencies in results. It is quite obvious from our findings that QT-DDIs and their monitoring protocols should be given appropriate consideration in clinical practice.

The prevalence of QT prolonging drugs and QT-DDIs in various types of cancer has not been the subject of studies conducted in past. These parameters were considered in the current study. We identified a high prevalence of QT prolonging drugs and QT-DDIs in breast cancer, gastrointestinal cancer, gynecologic cancer and non-hodgkin lymphoma. The high prevalence of QT-DDIs among cancer patients was due to the frequent use of the QT prolonging drugs. Appropriate considerations are needed to avoid any detrimental effects associated with QT interval prolonging drugs and QT-DDIs. We identified that the potential risk of QT-DDIs increases with rising number of all prescribed medications, QT interval prolonging drugs and supportive care drugs. The patients with breast cancer and gastrointestinal cancer are significantly exposed to QT-DDIs.

Concomitant use of QT prolonging drugs, possibly leading to fatal outcomes, should be avoided [26]. Several drugs involved in QT-DDIs represented a variety of therapeutic classes such as anticancer, antimicrobials, antiemetics and antipsychotics. QT-DDIs involving these drug classes potentiate the drug induced QTc prolongation and subsequent TdP. There is scarcity of information to guide physicians about the risks of QT-DDIs and this study would definitely help them about this critical area. It is difficult to guess the magnitude of knowledge of health care professionals about the use of QT drugs and QT-DDIs and whether or not they had made any attempts to avoid such drugs or their combinations.

One of the limitations of this study was the lack of ECG data. Consequently, we could not investigate the prevalence of the QTc interval prolongation among cancer patients. This is quite possible that these factors were not considered in routine clinical practice in oncology. In this study, Micromedex DrugReax® was used as a screening tool while other tools are also available and published literature have reported several inconsistencies among these tools [27].

## Conclusion

The present study shows a high prevalence of QT-DDIs in cancer patients. Various anticancer and supportive care drugs associated with QTc prolongation and TdP are often prescribed concomitantly in oncology, which may lead to lethal arrhythmias. Future studies should further explore the clinical outcomes of QT-DDIs such as QTc prolongation and TdP.

### Recommendations

The study findings suggest that the QT interval prolongation and subsequent risk of TdP should be considered as an essential component of the patients’ monitoring plan in the clinical practice. Moreover, an ECG should be done before starting a QT prolonging drug, 8–12 h after administration of QT prolonging drug or after increasing its dose, as recommended by American College of Cardiology Foundation (ACCF) and American Heart Association (AHA) [28]. The physicians should be aware of the arrhythmogenic risks associated with the QT interval prolonging drugs and QT-DDIs in oncology ward. In certain cases, where it is inevitable to avoid a QT prolonging drug or its combination, appropriate precautions such as ECG monitoring, dosage adjustment and rectifying the electrolyte imbalance should be undertaken to prevent the potential harmful consequences. The patients with breast cancer and gastrointestinal cancer are considerably exposed to the harmful effects of the QT-DDIs and need special attention. The QT-DDIs involving both the high-risk medications (known risk of TdP) should be particularly avoided. The updated drug information sources such as the AZCERT QT drugs lists [20] and the Micromedex DrugReax [19] can be helpful to clinicians regarding the drug selection in oncology.

## Additional files

Additional file 1: Table S1. Prevalence of QT prolonging drugs along with their TdP risks stratified with respect to various types of cancer. (PDF 234 kb)

Additional file 2: Table S2. Frequency of QT-DDIs along with their levels and TdP risks of drugs involved in these QT-DDIs stratified with respect to various types of cancer. (PDF 170 kb)

## Abbreviations

ACCF: American college of cardiology foundation
AHA: American heart association
ATC: Anatomical therapeutic chemical
ATH: Ayub teaching hospital
AZCERT: Arizona center for education and research on therapeutics
DDIs: Drug-drug interactions
ECG: Electrocardiogram
HMC: Hayatabad medical complex
QT-DDIs: QT prolonging drug-drug interactions
SPSS: Statistical package for the social sciences
TdP: Torsades de pointes
WHO: World health organization
