# Supplementary material for: Frequency, characteristics and risk factors of QT interval prolonging drugs and drug-drug interactions in cancer patients: a multicenter study
Source: BMC Pharmacol Toxicol. 2017 Dec 1;18:75. doi: 10.1186/s40360-017-0181-2 (PMC5710059; doi:10.1186/s40360-017-0181-2)
Supplement: Supplementary file 1 — Prevalence of QT prolonging drugs along with their TdP risks stratified with respect to various types of cancer. (PDF 234 kb) [file 40360_2017_181_MOESM1_ESM.pdf]

Supplementary Table S1: Prevalence of QT prolonging drugs along with their TdP risks stratified with respect to various types of cancer

Diagnoses	QT drugs	TdP risk	QT drugs: n (%)
**Breast cancer**	Ondansetron	Known risk of TdP	53
	Ciprofloxacin	Known risk of TdP	32
	Tropisetron	Possible risk of TdP	20
	Metoclopramide	Conditional risk of TdP	18
	Omeprazole	Conditional risk of TdP	8
	Esomeprazole	Conditional risk of TdP	6
	Capecitabine	Possible risk of TdP	4
	Tamoxifen	Possible risk of TdP	4
	Domperidone	Known risk of TdP	1
	Clarithromycin	Known risk of TdP	1
	Hydrochlorothiazide	Conditional risk of TdP	1
**Gastrointestinal cancer**	Ondansetron	Known risk of TdP	46
	Metoclopramide	Conditional risk of TdP	21
	Capecitabine	Possible risk of TdP	25
	Oxaliplatin	Known risk of TdP	20
	Tropisetron	Possible risk of TdP	17
	Omeprazole	Conditional risk of TdP	17
	Ciprofloxacin	Known risk of TdP	13
	Esomeprazole	Conditional risk of TdP	7
	Domperidone	Known risk of TdP	1
	Furosemide	Conditional risk of TdP	1
**Non hodgkin lymphoma**	Ondansetron	Known risk of TdP	39
	Tropisetron	Possible risk of TdP	33
	Metoclopramide	Conditional risk of TdP	25
	Esomeprazole	Conditional risk of TdP	16
	Omeprazole	Conditional risk of TdP	14
	Ciprofloxacin	Known risk of TdP	5
	Metronidazole	Conditional risk of TdP	3
	Domperidone	Known risk of TdP	3
	Furosemide	Conditional risk of TdP	3
	Amitriptyline	Conditional risk of TdP	1
	Promethazine	Possible risk of TdP	1
	Loperamide	Conditional risk of TdP	1
**Gynecologic cancer**	Ondansetron	Known risk of TdP	25
	Tropisetron	Possible risk of TdP	9
	Ciprofloxacin	Known risk of TdP	8
	Omeprazole	Conditional risk of TdP	7
	Metoclopramide	Conditional risk of TdP	7
	Esomeprazole	Conditional risk of TdP	5
	Domperidone	Known risk of TdP	3
	Metronidazole	Conditional risk of TdP	1
	Clarithromycin	Known risk of TdP	1
	Promethazine	Possible risk of TdP	1
**Genitourinary cancer**	Ondansetron	Known risk of TdP	18
	Esomeprazole	Conditional risk of TdP	5
	Domperidone	Known risk of TdP	4
	Metoclopramide	Conditional risk of TdP	4
	Tropisetron	Possible risk of TdP	3
	Furosemide	Conditional risk of TdP	2
	Ciprofloxacin	Known risk of TdP	1
	Metronidazole	Conditional risk of TdP	1
	Diphenhydramine	Conditional risk of TdP	1

**Acute lymphoblastic leukemia**	Metoclopramide	Conditional risk of TdP	16
	Omeprazole	Conditional risk of TdP	16
	Ondansetron	Known risk of TdP	7
	Metronidazole	Conditional risk of TdP	6
	Esomeprazole	Conditional risk of TdP	4
	Tropisetron	Possible risk of TdP	2
	Ciprofloxacin	Known risk of TdP	1
	Furosemide	Conditional risk of TdP	1
	Diphenhydramine	Conditional risk of TdP	1
	Fluconazole	Known risk of TdP	1
	Clarithromycin	Known risk of TdP	1
	Amitriptyline	Conditional risk of TdP	1
**Chronic lymphocytic leukemia**	Tropisetron	Possible risk of TdP	11
	Metoclopramide	Conditional risk of TdP	8
	Ciprofloxacin	Known risk of TdP	4
	Esomeprazole	Conditional risk of TdP	3
	Metronidazole	Conditional risk of TdP	3
	Fluconazole	Known risk of TdP	2
	Omeprazole	Conditional risk of TdP	2
	Domperidone	Known risk of TdP	1
	Furosemide	Conditional risk of TdP	1
	Amitriptyline	Conditional risk of TdP	1
	Norfloxacin	Possible risk of TdP	1
	Ondansetron	Known risk of TdP	1
**Musculoskeletal cancer**	Ondansetron	Known risk of TdP	9
	Metoclopramide	Conditional risk of TdP	7
	Tropisetron	Possible risk of TdP	6
	Ciprofloxacin	Known risk of TdP	4
	Omeprazole	Conditional risk of TdP	4
	Esomeprazole	Conditional risk of TdP	3
**Hodgkin lymphoma**	Ondansetron	Known risk of TdP	9
	Metoclopramide	Conditional risk of TdP	8
	Tropisetron	Possible risk of TdP	2
	Esomeprazole	Conditional risk of TdP	2
	Domperidone	Known risk of TdP	2
	Ciprofloxacin	Known risk of TdP	1
	Omeprazole	Conditional risk of TdP	1
**Colo rectal carcinoma**	Capecitabine	Possible risk of TdP	8
	Oxaliplatin	Known risk of TdP	7
	Ondansetron	Known risk of TdP	5
	Tropisetron	Possible risk of TdP	2
	Metoclopramide	Conditional risk of TdP	1
	Ciprofloxacin	Known risk of TdP	1
	Metronidazole	Conditional risk of TdP	1
	Furosemide	Conditional risk of TdP	1
	Fluconazole	Known risk of TdP	1
**Acute mylogenous leukemia**	Omeprazole	Conditional risk of TdP	7
	Metoclopramide	Conditional risk of TdP	5
	Metronidazole	Conditional risk of TdP	3
	Clarithromycin	Known risk of TdP	2
	Tropisetron	Possible risk of TdP	2
	Ondansetron	Known risk of TdP	2
	Diphenhydramine	Conditional risk of TdP	1
**Lung cancer**	Ondansetron	Known risk of TdP	6
	Tropisetron	Possible risk of TdP	3
	Metoclopramide	Conditional risk of TdP	1

	Omeprazole	Conditional risk of TdP	1
	Esomeprazole	Conditional risk of TdP	1
	Domperidone	Known risk of TdP	1
**Neurological cancer**	Ondansetron	Known risk of TdP	6
	Esomeprazole	Conditional risk of TdP	2
	Domperidone	Known risk of TdP	2
	Metoclopramide	Conditional risk of TdP	1
	Omeprazole	Conditional risk of TdP	1
**Adenocarcinoma**	Metoclopramide	Conditional risk of TdP	5
	Tropisetron	Possible risk of TdP	4
	Omeprazole	Conditional risk of TdP	4
	Ondansetron	Known risk of TdP	3
	Ciprofloxacin	Known risk of TdP	2
	Furosemide	Conditional risk of TdP	2
	Esomeprazole	Conditional risk of TdP	2
	Oxaliplatin	Known risk of TdP	1
	Hydrochlorothiazide	Conditional risk of TdP	1
**Head and neck cancer**	Ondansetron	Known risk of TdP	4
	Tropisetron	Possible risk of TdP	4
	Ciprofloxacin	Known risk of TdP	2
	Capecitabine	Possible risk of TdP	2
	Metoclopramide	Conditional risk of TdP	1
**Ovarian cancer**	Ondansetron	Known risk of TdP	4
	Tropisetron	Possible risk of TdP	4
	Domperidone	Known risk of TdP	1
	Diphenhydramine	Conditional risk of TdP	1
	Fluconazole	Known risk of TdP	1
	Tamoxifen	Possible risk of TdP	1
	Metoclopramide	Conditional risk of TdP	1
**Prostate cancer**	Metoclopramide	Conditional risk of TdP	4
	Metronidazole	Conditional risk of TdP	2
	Ketoconazole	Conditional risk of TdP	1
	Ciprofloxacin	Known risk of TdP	1
**Chronic myelogenous leukemia**	Metronidazole	Conditional risk of TdP	3
	Metoclopramide	Conditional risk of TdP	3
	Ciprofloxacin	Known risk of TdP	2
	Omeprazole	Conditional risk of TdP	2
	Nilotinib	Possible risk of TdP	1
	Ondansetron	Known risk of TdP	1
**Stomach cancer**	Tropisetron	Possible risk of TdP	3
	Ondansetron	Known risk of TdP	2
	Metoclopramide	Conditional risk of TdP	1
	Ciprofloxacin	Known risk of TdP	1
	Capecitabine	Possible risk of TdP	1
	Metronidazole	Conditional risk of TdP	1
	Amphotericin B	Conditional risk of TdP	1
	Levofloxacin	Known risk of TdP	1
**Malignant round blue cell tumor**	Ondansetron	Known risk of TdP	3
	Ciprofloxacin	Known risk of TdP	1
**Metastatic colo rectal carcinoma**	Capecitabine	Possible risk of TdP	3
	Ondansetron	Known risk of TdP	1
**Chronic lymphoid leukemia**	Ciprofloxacin	Known risk of TdP	2
	Metronidazole	Conditional risk of TdP	1
	Furosemide	Conditional risk of TdP	1
	Ondansetron	Known risk of TdP	1
**Acute myeloid leukemia**	Ondansetron	Known risk of TdP	2

	Metoclopramide	Conditional risk of TdP	1
	Ciprofloxacin	Known risk of TdP	1
	Domperidone	Known risk of TdP	1
	Furosemide	Conditional risk of TdP	1
	Ketoconazole	Conditional risk of TdP	1
**Carcinoma of unknown primary**	Ondansetron	Known risk of TdP	2
	Ciprofloxacin	Known risk of TdP	1
	Furosemide	Conditional risk of TdP	1
**Leiomyosarcoma**	Ondansetron	Known risk of TdP	2
**Multiple myeloma**	Metoclopramide	Conditional risk of TdP	2
	Tropisetron	Possible risk of TdP	1
	Furosemide	Conditional risk of TdP	1
	Diphenhydramine	Conditional risk of TdP	1
	Fluconazole	Known risk of TdP	1
	Fluoxetine	Conditional risk of TdP	1
**Plasma cell leukemia**	Ondansetron	Known risk of TdP	2
	Esomeprazole	Conditional risk of TdP	1
**Spindle cell carcinoma**	Ondansetron	Known risk of TdP	2
	Ciprofloxacin	Known risk of TdP	1
**Squamous cell carcinoma**	Metoclopramide	Conditional risk of TdP	2
	Tropisetron	Possible risk of TdP	1
	Ondansetron	Known risk of TdP	1
**Rhabdomyosarcoma**	Ondansetron	Known risk of TdP	2
	Metoclopramide	Conditional risk of TdP	1
	Tropisetron	Possible risk of TdP	1
**Uterine sarcoma**	Ondansetron	Known risk of TdP	2
	Metoclopramide	Conditional risk of TdP	1
	Amphotericin B	Conditional risk of TdP	1
**Brain tumor**	Ondansetron	Known risk of TdP	1
	Capecitabine	Possible risk of TdP	1
**Breast and ovary syndrome**	Tropisetron	Possible risk of TdP	1
	Esomeprazole	Conditional risk of TdP	1
**Cervical cancer**	Tropisetron	Possible risk of TdP	1
	Metronidazole	Conditional risk of TdP	1
**Chronic endometritis**	Ondansetron	Known risk of TdP	1
**Chronic myeloid leukemia**	Ciprofloxacin	Known risk of TdP	1
	Amphotericin B	Conditional risk of TdP	1
**Gall bladder cancer**	Ondansetron	Known risk of TdP	1
	Metoclopramide	Conditional risk of TdP	1
	Capecitabine	Possible risk of TdP	1
	Oxaliplatin	Known risk of TdP	1
**Germ cell testes**	Tropisetron	Possible risk of TdP	1
**Hairy cell leukemia**	Ondansetron	Known risk of TdP	1
	Esomeprazole	Conditional risk of TdP	1
**Hepatic tumor**	Furosemide	Conditional risk of TdP	1
**Idiopathic thrombocytopenia purpura**	Tropisetron	Possible risk of TdP	1
	Omeprazole	Conditional risk of TdP	1
**Liver metastasis**	Metoclopramide	Conditional risk of TdP	1
**Lymphophylloid disorder**	Ondansetron	Known risk of TdP	1
**Malignant Ascites**	Ondansetron	Known risk of TdP	1
**Malignant lymphoma**	Ondansetron	Known risk of TdP	1
**Malignant melanoma**	Metoclopramide	Conditional risk of TdP	1
	Tropisetron	Possible risk of TdP	1
**Mass in the head of pancreas**	Metoclopramide	Conditional risk of TdP	1
**Metastatic bone disease**	Ondansetron	Known risk of TdP	1
**Metastatic breast cancer**	Haloperidol	Known risk of TdP	1

**Metastatic cancer**	Ondansetron	Known risk of TdP	1
**Metastatic lung cancer**	Tropisetron	Possible risk of TdP	1
**Molar pregnancy**	Tropisetron	Possible risk of TdP	1
**Multiple bone degeneration**	Ondansetron	Known risk of TdP	1
	Metoclopramide	Conditional risk of TdP	1
	Domperidone	Known risk of TdP	1
	Diphenhydramine	Conditional risk of TdP	1
**Myelofibrosis**	Omeprazole	Conditional risk of TdP	1
	Furosemide	Conditional risk of TdP	1
	Moxifloxacin	Known risk of TdP	1
**Neuroendocrine tumor**	Ondansetron	Known risk of TdP	1
	Metoclopramide	Conditional risk of TdP	1
	Tropisetron	Possible risk of TdP	1
	Omeprazole	Conditional risk of TdP	1
	Furosemide	Conditional risk of TdP	1
	Hydrochlorothiazide	Conditional risk of TdP	1
**Non-small cell lung cancer**	Ondansetron	Known risk of TdP	1
**Osteosarcoma**	Tropisetron	Possible risk of TdP	1
**Pancreatic ductal carcinoma**	Tropisetron	Possible risk of TdP	1
**Prostate adenocarcinoma**	Leuprolide Acetate	Possible risk of TdP	1
**Rectal cancer**	Tropisetron	Possible risk of TdP	1
	Capecitabine	Possible risk of TdP	1
	Oxaliplatin	Known risk of TdP	1
**Respiratory cancer**	Ondansetron	Known risk of TdP	1
	Ciprofloxacin	Known risk of TdP	1
**Skin cancer**	Ondansetron	Known risk of TdP	1
	Metoclopramide	Conditional risk of TdP	1
**Spinal tumor (Metastatic carcinoma)**	Ciprofloxacin	Known risk of TdP	1
	Metronidazole	Conditional risk of TdP	1
**Spindle cell neoplasm**	Ondansetron	Known risk of TdP	1
	Ciprofloxacin	Known risk of TdP	1
**Thyroid cancer**	Metoclopramide	Conditional risk of TdP	1
**Transitional cell carcinoma of bladder**	Ciprofloxacin	Known risk of TdP	1
**Undifferentiated malignant neoplasm**	Ondansetron	Known risk of TdP	1
**Uterine fundus**	Ondansetron	Known risk of TdP	1
