# Supplementary material for: Frequency, characteristics and risk factors of QT interval prolonging drugs and drug-drug interactions in cancer patients: a multicenter study
Source: BMC Pharmacol Toxicol. 2017 Dec 1;18:75. doi: 10.1186/s40360-017-0181-2 (PMC5710059; doi:10.1186/s40360-017-0181-2)
Supplement: Supplementary file 2 — Frequency of QT-DDIs along with their levels and TdP risks of drugs involved in these QT-DDIs stratified with respect to various types of cancer. (PDF 170 kb) [file 40360_2017_181_MOESM2_ESM.pdf]

Supplementary Table S2: Frequency of QT-DDIs along with their levels and TdP risks of drugs involved in these QT-DDIs stratified with respect to various types of cancer

Diagnoses	QT-DDI	TdP risk		Levels of QT-DDIs		Frequency
		Drug 1	Drug 2	Severity	Documentation	QT-DDIs: n
**Breast cancer**	Ondansetron-Prochlorperazine	Known risk of TdP	Not included in lists	Major	Fair	31
	Ciprofloxacin-Ondansetron	Known risk of TdP	Known risk of TdP	Major	Fair	31
	Ciprofloxacin-Prochlorperazine	Known risk of TdP	Not included in lists	Major	Fair	31
	Dolasetron-Metronidazole	Possible risk of TdP	Conditional risk of TdP	Major	Fair	1
	Ciprofloxacin-Dolasetron	Known risk of TdP	Possible risk of TdP	Major	Fair	1
	Ciprofloxacin-Metronidazole	Known risk of TdP	Conditional risk of TdP	Major	Fair	1
**Gastrointestinal cancer**	Ondansetron-Prochlorperazine	Known risk of TdP	Not included in lists	Major	Fair	26
	Ciprofloxacin-Ondansetron	Known risk of TdP	Known risk of TdP	Major	Fair	13
	Ciprofloxacin-Prochlorperazine	Known risk of TdP	Not included in lists	Major	Fair	12
	Dolasetron-Ondansetron	Possible risk of TdP	Known risk of TdP	Major	Fair	2
	Ciprofloxacin-Dolasetron	Known risk of TdP	Possible risk of TdP	Major	Fair	1
**Gynecologic cancer**	Ciprofloxacin-Ondansetron	Known risk of TdP	Known risk of TdP	Major	Fair	8
	Ciprofloxacin-Prochlorperazine	Known risk of TdP	Not included in lists	Major	Fair	6
	Ondansetron-Prochlorperazine	Known risk of TdP	Not included in lists	Major	Fair	6
	Ciprofloxacin-Promethazine	Known risk of TdP	Possible risk of TdP	Major	Fair	1
	Clarithromycin-Dolasetron	Known risk of TdP	Possible risk of TdP	Major	Fair	1
	Dolasetron-metronidazole	Possible risk of TdP	Conditional risk of TdP	Major	Fair	1
	Dolasetron-ondansetron	Possible risk of TdP	Known risk of TdP	Major	Fair	1
	Ondansetron-Promethazine	Known risk of TdP	Conditional risk of TdP	Major	Fair	1
	Prochlorperazine-Promethazine	Not included in lists	Possible risk of TdP	Major	Fair	1
	Ciprofloxacin-Dolasetron	Known risk of TdP	Possible risk of TdP	Major	Fair	1
	Ciprofloxacin-Metronidazole	Known risk of TdP	Conditional risk of TdP	Major	Fair	1
**Genitourinary cancer**	Ondansetron-Prochlorperazine	Known risk of TdP	Not included in lists	Major	Fair	7
	Ciprofloxacin-Ondansetron	Known risk of TdP	Known risk of TdP	Major	Fair	1
	Ciprofloxacin-Prochlorperazine	Known risk of TdP	Not included in lists	Major	Fair	1
	Metronidazole-Ondansetron	Conditional risk of TdP	Known risk of TdP	Major	Fair	1
	Metronidazole-Prochlorperazine	Conditional risk of TdP	Not included in lists	Major	Fair	1
**Musculoskeletal cancer**	Ciprofloxacin-Ondansetron	Known risk of TdP	Known risk of TdP	Major	Fair	4
	Ciprofloxacin-Prochlorperazine	Known risk of TdP	Not included in lists	Major	Fair	4
	Ondansetron-Prochlorperazine	Known risk of TdP	Not included in lists	Major	Fair	4
	Dolasetron-Ondansetron	Possible risk of TdP	Known risk of TdP	Major	Fair	1
**Chronic**	Dolasetron-Metronidazole	Possible risk of TdP	Conditional risk of TdP	Major	Fair	3

**lymphocytic leukemia**	Ciprofloxacin-Dolasetron	Known risk of TdP	Possible risk of TdP	Major	Fair	2
	Dolasetron-Norfloxacin	Possible risk of TdP	Possible risk of TdP	Major	Fair	1
	Fluconazole -- Metronidazole	Known risk of TdP	Conditional risk of TdP	Major	Fair	1
	Fluconazole-Sulfamethoxazole/ Trimethoprim	Known risk of TdP	Not included in lists	Major	Fair	1
	Metronidazole-Norfloxacin	Conditional risk of TdP	Possible risk of TdP	Major	Fair	1
	Ciprofloxacin-Fluconazole	Known risk of TdP	Known risk of TdP	Major	Fair	1
	Ciprofloxacin-Metronidazole	Known risk of TdP	Conditional risk of TdP	Major	Fair	1
	Dolasetron-Fluconazole	Possible risk of TdP	Known risk of TdP	Major	Fair	1
**Neurological cancer**	Ondansetron-Prochlorperazine	Known risk of TdP	Not included in lists	Major	Fair	3
	Prochlorperazine-Sulfamethoxazole	Not included in lists	Not included in lists	Major	Fair	1
	Prochlorperazine-Trimethoprim	Not included in lists	Not included in lists	Major	Fair	1
**Head and neck cancer**	Ciprofloxacin-ondansetron	Known risk of TdP	Known risk of TdP	Major	Fair	2
	Ciprofloxacin-Prochlorperazine	Known risk of TdP	Not included in lists	Major	Fair	2
	Ondansetron-Prochlorperazine	Known risk of TdP	Not included in lists	Major	Fair	2
**Adenocarcinoma**	Ciprofloxacin-Ondansetron	Known risk of TdP	Known risk of TdP	Major	Fair	2
	Ciprofloxacin-Prochlorperazine	Known risk of TdP	Not included in lists	Major	Fair	2
	Ondansetron-Prochlorperazine	Known risk of TdP	Not included in lists	Major	Fair	2
	Dolasetron-Octreotide	Possible risk of TdP	Not included in lists	Major	Fair	1
	Dolasetron-Ondansetron	Possible risk of TdP	Known risk of TdP	Major	Fair	1
**Non hodgkin lymphoma**	Ciprofloxacin-Metronidazole	Known risk of TdP	Conditional risk of TdP	Major	Fair	2
	Ciprofloxacin-Ondansetron	Known risk of TdP	Known risk of TdP	Major	Fair	2
	Ondansetron-Prochlorperazine	Known risk of TdP	Not included in lists	Major	Fair	2
	Ondansetron-Promethazine	Known risk of TdP	Conditional risk of TdP	Major	Fair	1
	Prochlorperazine-Promethazine	Not included in lists	Possible risk of TdP	Major	Fair	1
	Ciprofloxacin-Prochlorperazine	Known risk of TdP	Not included in lists	Major	Fair	1
	Metronidazole-Ondansetron	Conditional risk of TdP	Known risk of TdP	Major	Fair	1
**Chronic myelogenous leukemia**	Metronidazole-Nilotinib	Conditional risk of TdP	Conditional risk of TdP	Major	Fair	2
	Metronidazole-Ondansetron	Conditional risk of TdP	Known risk of TdP	Major	Fair	1
	Metronidazole-Tizanidine	Conditional risk of TdP	Not included in lists	Major	Fair	1
	Nilotinib-Tizanidine	Conditional risk of TdP	Not included in lists	Major	Fair	1
	Ciprofloxacin-Metronidazole	Known risk of TdP	Conditional risk of TdP	Major	Fair	1
	Ciprofloxacin-Ondansetron	Known risk of TdP	Known risk of TdP	Major	Fair	1
**Acute lymphoblastic leukemia**	Amitriptyline-Ondansetron	Conditional risk of TdP	Possible risk of TdP	Major	Fair	1
	Ciprofloxacin-Dolasetron	Known risk of TdP	Possible risk of TdP	Major	Fair	1
	Clarithromycin-Metronidazole	Known risk of TdP	Conditional risk of TdP	Major	Fair	1
	Clarithromycin-Ondansetron	Known risk of TdP	Known risk of TdP	Major	Fair	1
	Fluconazole-Metronidazole	Known risk of TdP	Conditional risk of TdP	Major	Fair	1
	Metronidazole-Ondansetron	Conditional risk of TdP	Known risk of TdP	Major	Fair	1

**Acute myeloid leukemia**	Ciprofloxacin-Ketoconazole	Known risk of TdP	Conditional risk of TdP	Major	Fair	1
**Acute mylogenous leukemia**	Clarithromycin-Ondansetron	Known risk of TdP	Known risk of TdP	Major	Fair	1
**Carcinoma of unknown primary**	Ciprofloxacin-Ondansetron	Known risk of TdP	Known risk of TdP	Major	Fair	1
	Ciprofloxacin-Prochlorperazine	Known risk of TdP	Not included in lists	Major	Fair	1
	Ondansetron-Prochlorperazine	Known risk of TdP	Not included in lists	Major	Fair	1
**Chronic lymphoid leukemia**	Ciprofloxacin-Metronidazole	Known risk of TdP	Conditional risk of TdP	Major	Fair	1
	Ciprofloxacin-Ondansetron	Known risk of TdP	Known risk of TdP	Major	Fair	1
**Colo rectal carcinoma**	Ciprofloxacin-Fluconazole	Known risk of TdP	Known risk of TdP	Major	Fair	1
	Ciprofloxacin-Metronidazole	Known risk of TdP	Conditional risk of TdP	Major	Fair	1
	Fluconazole-Metronidazole	Known risk of TdP	Conditional risk of TdP	Major	Fair	1
**Lung cancer**	Ciprofloxacin-Ondansetron	Known risk of TdP	Known risk of TdP	Major	Fair	1
	Ciprofloxacin-Prochlorperazine	Known risk of TdP	Not included in lists	Major	Fair	1
	Ondansetron-Prochlorperazine	Known risk of TdP	Not included in lists	Major	Fair	1
**Malignant round blue cell tumor**	Ciprofloxacin-Ondansetron	Known risk of TdP	Known risk of TdP	Major	Fair	1
	Ondansetron-Tizanidine	Known risk of TdP	Not included in lists	Major	Fair	1
**Multiple myeloma**	Fluoxetine-Fluconazole	Conditional risk of TdP	Known risk of TdP	Major	Fair	1
**Neuroendocrine tumor**	Dolasetron-Octreotide	Possible risk of TdP	Not included in lists	Major	Fair	1
**Prostate cancer**	Ciprofloxacin-Ketoconazole	Known risk of TdP	Conditional risk of TdP	Major	Fair	1
	Ciprofloxacin-Metronidazole	Known risk of TdP	Conditional risk of TdP	Major	Fair	1
	Ketoconazole-Metronidazole	Known risk of TdP	Conditional risk of TdP	Major	Fair	1
**Respiratory cancer**	Ciprofloxacin-Ondansetron	Known risk of TdP	Known risk of TdP	Major	Fair	1
	Ciprofloxacin-Prochlorperazine	Known risk of TdP	Not included in lists	Major	Fair	1
	Ondansetron-Prochlorperazine	Known risk of TdP	Not included in lists	Major	Fair	1
**Spindle cell carcinoma**	Ciprofloxacin-Ondansetron	Known risk of TdP	Known risk of TdP	Major	Fair	1
	Ciprofloxacin-Prochlorperazine	Known risk of TdP	Not included in lists	Major	Fair	1
	Ondansetron-Prochlorperazine	Known risk of TdP	Not included in lists	Major	Fair	1
**Spindle cell neoplasm**	Ciprofloxacin-Ondansetron	Known risk of TdP	Known risk of TdP	Major	Fair	1
	Ciprofloxacin-Prochlorperazine	Known risk of TdP	Not included in lists	Major	Fair	1
	Ondansetron-Prochlorperazine	Known risk of TdP	Not included in lists	Major	Fair	1
**Stomach cancer**	Ciprofloxacin-Levofloxacin	Known risk of TdP	Known risk of TdP	Major	Fair	1
	Ciprofloxacin-Metronidazole	Known risk of TdP	Conditional risk of TdP	Major	Fair	1
	Levofloxacin-Metronidazole	Known risk of TdP	Conditional risk of TdP	Major	Fair	1
